# Sulfated Polysaccharides from Macroalgae—A Simple Roadmap for Chemical Characterization

**DOI:** 10.3390/polym15020399

**Published:** 2023-01-12

**Authors:** Alice Martins, Celso Alves, Joana Silva, Susete Pinteus, Helena Gaspar, Rui Pedrosa

**Affiliations:** 1 MARE—Marine and Environmental Sciences Centre/ARNET-Aquatic Research Network, Polytechnic of Leiria, 2520-630 Peniche, Portugal; 2 BioISI-Biosystems and Integrative Sciences Institute, Faculty of Sciences, University of Lisbon, 1749-016 Lisboa, Portugal; 3 MARE/ARNET/ESTM, Polytechnic of Leiria, 2520-614 Peniche, Portugal

**Keywords:** seaweeds, marine natural products, sulfated polymers, chemical analysis

## Abstract

The marine environment presents itself as a treasure chest, full of a vast diversity of organisms yet to be explored. Among these organisms, macroalgae stand out as a major source of natural products due to their nature as primary producers and relevance in the sustainability of marine ecosystems. Sulfated polysaccharides (SPs) are a group of polymers biosynthesized by macroalgae, making up part of their cell wall composition. Such compounds are characterized by the presence of sulfate groups and a great structural diversity among the different classes of macroalgae, providing interesting biotechnological and therapeutical applications. However, due to the high complexity of these macromolecules, their chemical characterization is a huge challenge, driving the use of complementary physicochemical techniques to achieve an accurate structural elucidation. This review compiles the reports (2016–2021) of state-of-the-art methodologies used in the chemical characterization of macroalgae SPs aiming to provide, in a simple way, a key tool for researchers focused on the structural elucidation of these important marine macromolecules.

## 1. Introduction

Polysaccharides are condensate polymers of various sugars, which themselves are cyclic ethers that contain, typically, many hydroxy (–OH) substituents and, in some cases, other substituents such as amines and carboxylic acid groups. There are so many sugar monomers, and the diversity of polysaccharides is so broad, that it is not possible to write a single general structure as it is commonly done for proteins and nucleic acids.

The versatility of marine polysaccharides, e.g., their abundance, biodegradability, and biocompatibility, has been extensively investigated in the pharmaceutical and biomedical fields due to their wide range of therapeutic properties as antitumoral, anti-inflammatory, immunomodulatory, antimicrobial, and drug-release applications [1,2]. Additionally, these natural polymers are also reported for their cosmeceutical and nutraceutical potential [3], being increasingly explored by the cosmetic, food, and feed industries. Therefore, efforts focused on the elucidation of their accurate chemical structure are very important to establish a rational structure-bioactivity relationship.

## 2. Chemical Features of Macroalgae Sulfated Polysaccharides

**Figure 1:**
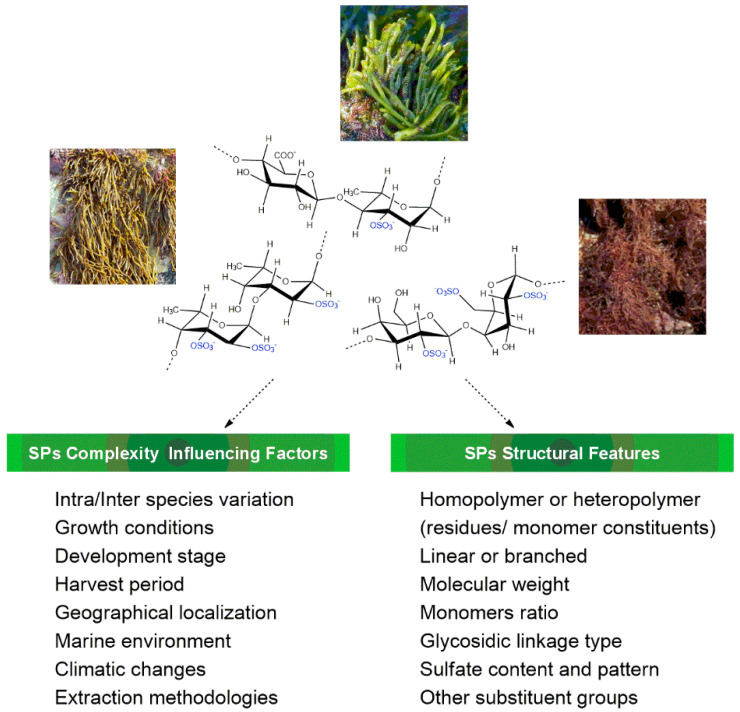
Features related to macroalgae sulfated polysaccharides’ complexity.

As fully reported, macroalgae are known to be a good source of a variety of sulfated polysaccharides (SPs), with their bioactivities being influenced by their chemical structure [4,5,6]. However, a complete and unequivocal chemical characterization of SPs continues to be a challenge due to their structural complexity: type of polymer (homo/heteropolymer, linear/branched), molecular weight (MW), sugar composition, type of *O*-glycosidic linkage, sulfate pattern, and other substituents (e.g., acetate, pyruvate). These structural features strongly depend on a set of biotic and abiotic factors (Figure 1), such as macroalgae species, growth stage, harvest season, marine environment, climatic changes, geographical localization, and extraction/purification methodologies, which, taken together, also contribute to make SPs’ structural elucidation a very difficult task [7,8,9].

**Figure 2:**
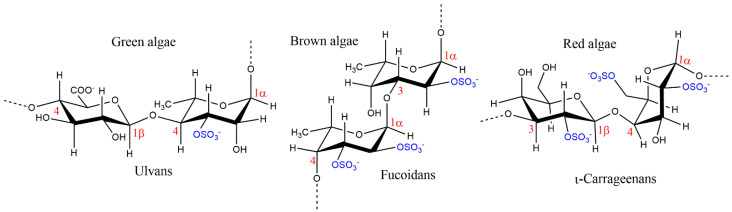
Characteristic backbones of macroalgae sulfated polysaccharides.

The extensive reviews reported in the literature [8,9,10,11,12,13,14,15,16,17,18,19] on the structural features of macroalgae SPs reveal that, despite their chemical structural variability, some similar backbones are characteristic of each seaweed phyllo. The most simple and representative structural backbones of the SPs biosynthesized by brown, red, and green macroalgae are depicted in Figure 2.

Fucoidans are the main SPs biosynthesized by brown algae. Besides fucose, the predominant sugar, other monomers such as glucose, galactose, xylose, mannose, and glucuronic acid also make up part of fucoidans’ structure. This group of SPs can be divided into two subgroups, one composed by alternating 1,3- and 1,4-linked α-l-fucopyranose residues and the other by α-1,3-l-fucopyranose, being sulfate groups linked to *O*-2 and/or *O*-3 and/or *O*-4 positions of fucose [4,13,16,17]. Fucoidans can be differentiated into several distinct groups according to the macroalgae species from which they are isolated, showing significant differences on their polydispersity behavior derived from a broad range of molecular weights, sugar, sulfate, and acetate contents, while enhanced bio-functional properties are achieved via structural modification of those SPs [9].

Carrageenans are the main characteristic SPs of red macroalgae and are conventionally categorized into six basic forms depending on their amount and position of sulfate groups, the number of 3,6-anhydrogalactose residues, source of extraction, and solubility, as: Kappa (κ)-, Iota (ɩ)-, Lambda (λ)-, Mu (μ)-, Nu (ν)-, and Theta (θ)-carrageenans. They are composed by alternating α-1,4-d-galactopyranose and β-1,3-d-galactopyranose (μ-, ν-, and λ-carrageenan) or by alternating β-1,3-d-galactopyranose and 3,6-anhydro-α-d-galactopyranose (κ-, ɩ-, and θ-carrageenan) [17,20]. Of these, κ, ɩ, and λ are of commercial importance due to their viscoelastic and gelling properties [10]. Due to their biocompatibility, emulsifying, thickening, gelling, and stabilizing abilities, they have several industrial applications, especially in the food, pharmaceutical, and cosmetic industries [21]. An example of a successful history is Carragelose^®^, an antiviral nasal spray that contains the linear SPs ɩ-carrageenan extracted from red edible seaweeds and is marketed as an over the counter (OTC) drug [22]. Due to the chemical properties of carrageenan-based hydrogels, these SPs are currently promising candidates for tissue engineering and regenerative medicine due to their similarity with native glycosaminoglycans [20].

Agar is a mixture of agarose and agaropectin consisting of d-galactose and 3,6-anhydro-α-l-galactose units joined by β-1,3- and α-1,4-glycosidic linkages. Sulfate and methoxyl groups, as well as pyruvic and d-guluronic acids, can be found in agar backbone [17]. Porphyrans and funorans, also known as agaroids, have a chemical structure very close to agars and are found in some species of red algae [16,23].

Ulvans and sulfated galactans are the main SPs found in green algae. Ulvans are water-soluble polyanionic heteropolysaccharides, with the ulvan backbone being frequently made of α- and β-(1,4)-linked monosaccharides (rhamnose, xylose, glucuronic, and iduronic acids) with characteristic repeating disaccharide units [16,17]. However, other monosaccharides are often reported in their composition, e.g., glucose, galactose, arabinose, and mannose [14]. Sulfated galactans are highly branched sulfated β-d-galactose molecules with (1,3) and (1,6) linkages, with sulfation mainly occurring at C-4 and C-6 positions [23].

Glycosaminoglycans (GAGs) are linear and heterogeneous sulfated glycans that can be found not only in green but also in red algae [13]. The skeletons of these polysaccharides are constituted by repeated building blocks of disaccharides composed of alternating uronic acid (UroA) or galactose (Gal) and hexosamine. The hexosamine may be glucosamine (GlcN) or *N*-acetylgalactosamine (GalNAc) and its differently substituted (mostly sulfated) derivatives. UroA can be either glucuronic acid (GlcA) or iduronic acid [13].

Some of these structural features are strictly linked with the selected extraction, depolymerization, and purification processes, which can be chosen according to the available technologies and therapeutic/industrial applications.

## 3. Extraction, Depolymerization, and Purification Processes

Different extraction/purification techniques employed to obtain polysaccharide-enriched products from macroalgae, and their pros and cons, were recently reviewed [6,14,17,24,25,26,27,28,29]. The chosen isolation procedure can strongly influence the molecular weight, monosaccharide composition, and sulfate content of SPs [28]. Although conventional extraction (CE) procedures (e.g., extraction with water in basic or acidic conditions at different temperatures) continue to be used, advanced extraction techniques such as subcritical water extraction (SWE), supercritical fluid extraction (SFE), microwave-assisted extraction (MAE), ultrasound-assisted extraction (UAE), pressurized liquid extraction (PLE), and enzymatic-assisted extraction (EAE) constitute efficient alternatives. Additionally, Matos et al. [29] reported the use of pulsed electric field (PEF) and ohmic heating (OH) as examples of promising and attractive electro-technologies to recover added-value compounds from macroalgae.

Since sulfated polysaccharides are complex macromolecules of high molecular weights, it is hard to achieve unequivocal structural characterization of intact polymers. Therefore, they need to be transformed into small oligomers and/or sugar monomers to facilitate further structural elucidation. Usually, the first step is the depolymerization, which can be achieved through acid (HCl, TFA, H_2_SO_4_), enzymatic (Celluclast, Viscozyme, Fucoidanase, etc.), or by high-pressure hydrolysis methods. In the following, the fractionation/purification steps of SPs’ hydrolysates can be performed with complementary methods: (i) physicochemical (precipitation, ultracentrifugation, complexation), (ii) membrane separation (dialysis, ultrafiltration), and (iii) chromatographic (ion-exchange chromatography (IEC) and size-exclusion chromatography (SEC), also referred to as gel permeation chromatography (GPC)). SPs are negatively charged molecules due to the presence of sulfate ions, and thus anion-exchange chromatography is very useful to eliminate neutral polymers, while size-exclusion chromatography allows measurements of total and molecular mass distributions. Therefore, the use of diethylaminoethyl anion-exchange (DEAE) chromatography, such as DEAE-Sepharose or DEAE-cellulose, is fully reported for SPs’ purification purposes and can be combined with SEC. More specific details regarding purification methodologies applied to polysaccharides from macroalgae and other natural sources were recently reviewed [6,30,31].

## 4. Chemical Characterization

The first approach aiming at the chemical characterization of macroalgae-derived SPs after extraction, fractionation, and/or purification procedures is the determination of the total content of carbohydrates, sulfates, and eventually other components, mostly proteins and phenolics, by using standard analytical methods.

The phenol-sulfuric acid method is the most used to estimate the concentration of total carbohydrates. The basic principle of the phenol-H_2_SO_4_ reaction established by Dubois et al. [32] is that carbohydrates, when dehydrated by reaction with concentrated sulfuric acid, produce furfural derivatives, which react with phenol, developing colored products [33]. d-glucose is widely used as a standard to obtain a calibration curve.

Sulfate content can be estimated by turbidimetric, colorimetric, and/or gravimetric methods. Turbidimetric methods, such as the gelatin-barium assay, quantify sulfate content on polysaccharide-enriched samples and are based on the reaction of the sulfate ion (SO_4_^2−^) with the barium ion (Ba^2+^), originating barium sulfate (BaSO_4_), a water-insoluble precipitate at a low pH. The turbidity generated by the precipitate is commonly established by gelatin [34,35,36]. The quantification through colorimetric assays is preceded by the polysaccharide hydrolysis and can be accomplished by using Azure A dye, which is able to bind to sulfate groups [37]. Sodium sulfate is widely used as a standard. The method of precipitation and weighing of sulfate as BaSO_4_ according to AOAC [38] is a widely used gravimetric method to determine the sulfate content.

The presence of proteins on crude SPs’ fractions can be estimated by the methods developed by Bradford [39], Spector [40], and/or Lowry et al. [41], while the total phenolic content can be evaluated by the Folin-Ciocalteu method. For each determination, bovine serum albumin and gallic acid can be used as standards, respectively.

**Figure 3:**
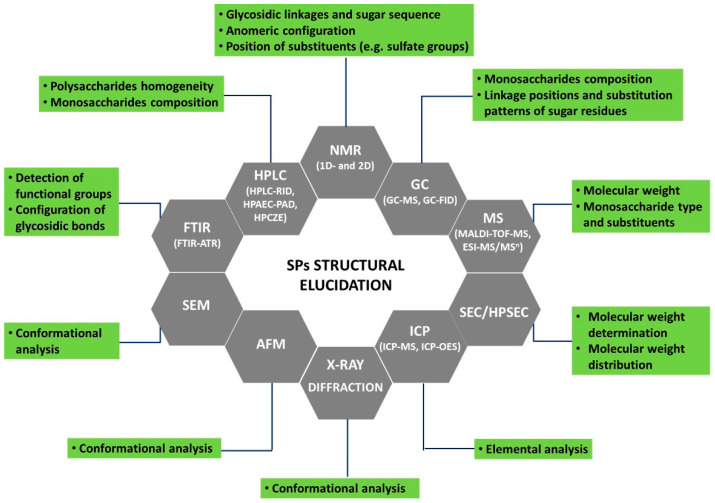
Current techniques for macroalgae sulfated polysaccharides’ structural characterization.

Besides the general component analysis usually performed on crude SPs (total carbohydrates, total protein, total phenolics, and total sulfate contents), more refined techniques need to be used to determine SPs’ chemical structural features. As reported by several authors [6,14,29], the elucidation of polysaccharides’ structure is a hard task due to the presence of multiple monosaccharide constituents, a variety of *O*-glycosidic linkages, high molecular weights, sugars’ branching, variable degrees of sulfation and substitution patterns, stereochemistry, as well as complex macromolecular properties as their aggregation modes. Effectively, to achieve a consistent structural characterization of these natural sugar polymers, it will be necessary to resort to several complementary analytical techniques to be applied to crude SPs and their derived hydrolysates. The most used techniques, and relevant information to be attained from each one, are summarized in Figure 3. Additionally, a set of chemical derivatization methods (methylation, periodate oxidation, etc.) coupled with those instrumental techniques can provide some insights into SPs’ chain structure.

Spectroscopy techniques such as Fourier transform infrared spectroscopy (FTIR), Fourier transform infrared spectroscopy-attenuated total reflection (FTIR-ATR), and Raman spectroscopy allow the detection of characteristic functional groups of SPs and can also provide some information regarding the type of glycosidic linkages. The anomeric configuration, sugar sequence, as well as the position of substituents, e.g., sulfate groups, can be determined by nuclear magnetic resonance (NMR) spectroscopy (1D and 2D experiments).

The determination of the average molecular weight (MW) and molecular weight distribution of SPs can be achieved through size-exclusion chromatography (SEC), while HPLC-SEC also offers high resolution and reproducibility and can simultaneously detect the homogeneity of polysaccharides. Refractive index (RI) and evaporative light scattering (ELSD) are the most common detectors coupled with SEC, but in some applications, multiangle laser light scattering (MALLS) is also used. The SEC-MALLS has the advantage to provide both molar mass and size independently of reference standards. Mass spectrometry techniques such as matrix-assisted laser desorption/ionization-time-of-flight mass spectrometry (MALDI-TOF-MS) and electrospray ionization tandem mass spectrometry (ESI-MS/MS^n^) are used to analyze macromolecules, including SPs, providing information not only about MW but also regarding monosaccharide type and substituents.

After hydrolysis, monosaccharides’ composition can be determined by gas chromatography coupled to mass spectrometry or to flame ionization detectors (GC-MS, GC-FID), high-performance liquid chromatography-refractive index detector (HPLC-RID), and high-performance anion-exchange chromatography combined with pulsed amperometric detection (HPAEC-PAD), as well as by high-performance capillary zone electrophoresis (HPCZE). GC analysis requires the conversion of sugars to volatile analogues such as alditol acetates, methyl, or trimethylsilyl derivatives, also providing information on the linkage positions and substitution patterns of constituent sugars.

Inductively coupled plasma-mass spectrometry (ICP-MS) or inductively coupled plasma-optical emission spectroscopy (ICP-OES) can be used to perform SPs’ elemental analysis. Other complementary techniques such as scanning electron microscopy (SEM), atomic force microscopy (AFM), X-ray diffraction (XRD), and circular dichroism (CD) can provide insights regarding the conformational analysis of SPs. More details about the above-outlined techniques were previously described [6,31]. Additionally, Table 1 compiles the methodologies used to attain the structural elucidation of SPs from brown, red, and green macroalgae over the last five years.

From the analysis of Table 1, it is evident that, besides the determination of total components (carbohydrates, sulfates, proteins, phenolics, glucuronic acid) and elemental (C, H, O, S) analysis, spectroscopic (FTIR, NMR) and chromatographic techniques (HPLC, GC, SEC, AEC) coupled to different detectors (MS, MALLS, RI, PAD, ELSD) are the most used techniques to attain the structural elucidation of SPs from macroalgae.

Examples of the application of several complementary techniques aiming at the structural elucidation of these marine macromolecules is evidenced by the work of Cao et al. [92] and Wahlström et al. [101]. Besides chemical modifications (acid hydrolysis, desulfation, methylation), Cao et al. [92] used HPAEC, HPGPC, FTIR, HILIC-FT-MS, GC-MS, and 1D- and 2D-NMR to perform the chemical characterization of SPs isolated from the green macroalgae *Monostroma nitidum,* while Wahlström et al. [101] have performed elemental analysis, FTIR, SEC, TGA, SEM, NMR, and HPAEC-PAD to characterize the SPs from *Ulva* spp.

**Figure 4:**
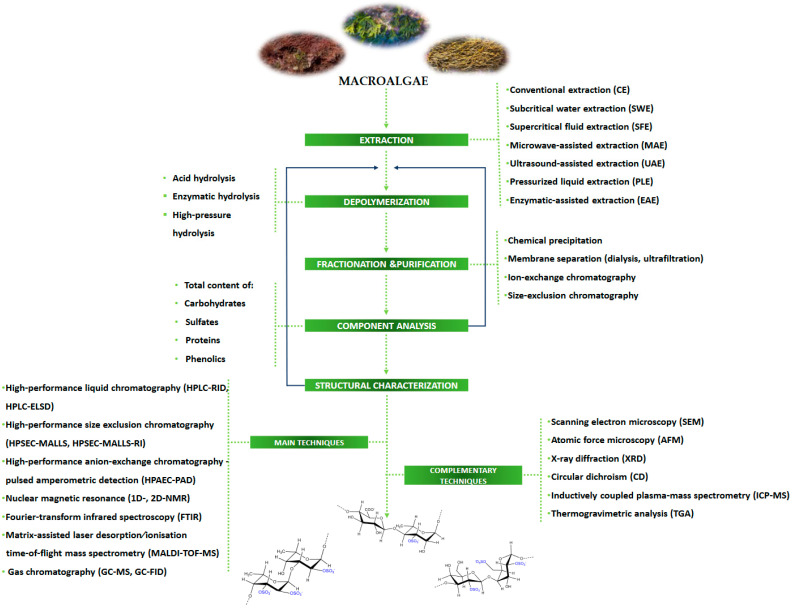
Roadmap of techniques/approaches for the chemical characterization of sulfated polysaccharides.

A general roadmap of the main steps and techniques and/or methods currently used for extraction and chemical characterization of sulfated polysaccharides from macroalgae is summarized in Figure 4.

## 5. Conclusions and Further Directions

Over the last years, sulfated polysaccharides have aroused the interest of the research community due to their broad applications in biomedical, functional food, and technological areas. However, the widespread use of these macromolecules remains a challenge, mainly due to different factors that, directly and/or indirectly, affect their unequivocal chemical characterization, such as seasonality, macroalgae species, SPs’ structural and conformational variability, high molecular weights, etc., influencing their bioavailability and physicochemical behavior. Effectively, the diversity and chemical complexity of these natural polymers make their structural elucidation a hard task. Several strategies have been used to characterize SPs and it is very clear that only the integration of distinct methodologies/techniques will provide complementary information that will allow researchers to build on the puzzle of SPs’ structure. This work also evidenced the need for a set of highly costly equipment, many of them only available in a few research institutions. These constraints highlight the importance of strengthening and stimulating collaborative networks between scientists for the development of new advanced tools and strategies to reach the most accurate chemical characterization of SPs extracted from natural resources.

## Figures and Tables

**Table 1:** Strategies for chemical characterization of sulfated polysaccharides isolated from macroalgae adopted in the last five years (2016–2021).

Algae	Source	Compound	Chemical Characterization	Reference
**Ochrophyta (Brown Algae)**				
*Chnoospora minima*	Southern coastal area of Sri Lanka	Fucoidan	Component analysis DEAE-Sepharose chromatography FTIR AGE HPAE-PAD NMR	[42]
*Cladosiphon okamuranus*	Ishigaki Island (Okinawa, Japan)	Fucoidan	Component analysis IEC GC-FID GC-MS Chemical modifications	[4]
*Dictyota bartayesiana* *Turbinaria decurrens*	Mandapam Coastal region, Rameswaram, Tamil Nadu, India	Fucoidan	Component analysis FTIR RP-HPLC DEAE-Cellulose chromatography Chemical modifications	[43]
*Ecklonia maxima*	HIK-Abalone Farm, Hermanus, South Africa	Fucoidan	Component analysis Ultracentrifugation FTIR NMR XRD	[44]
*Fucus evanescens*	-	Fucoidan	Component analysis SEC IEC NMR	[45]
*Himanthalia elongata*	Spanish Atlantic coasts (local supplier Porto-Muiños, A Coruña, Spain)	Fucoidans	FTIR HPSEC GC-FID	[46]
*Hizikia fusiforme*	-	Fucoidan	Component analysis HPGPC HPAEC-PAD FTIR NMR	[47]
*Kjellmaniella crassifolia*	Coast of Dalian, China	Fucoidans	Component analysis HPLC DEAE-Sepharose chromatography SEC FTIR 1D and 2D NMR	[48]
*Laminaria hyperborea*	Northeast Atlantic Ocean, Scandinavia	Sulfated fucans	AEC Raman spectroscopy ICP-MS HPSEC-MALLS	[49]
*Laminaria japonica*	Putian, Fujian, China	Fucoidans	DEAE-Cellulose chromatography Ultrafiltration Chemical modifications GC	[50]
*Laminaria japonica*	Crude commercial fucoidan (Rizhao Jiejing Ocean Biotechnology Development Co., Ltd., Rizhao, China)	Fucoidan	Elemental analysis Component analysis Chemical modifications 1D and 2D NMR GC-FID GC-MS SLS/DLS measurements FTIR AFM	[51]
*Lessonia* sp.	Tekenika Bay, Southern Chile	Sulfated fucan	FTIR NMR GC-FID DEAE HPLC	[52]
*Nizamuddinia zanardinii*	Rocky beaches of Chabahr at Oman Sea, South of Iran	Fucoidan	Component analysis FTIR GCMS HPSEC-UV-MALLS-RI SEM	[53]
*Padina commersonii*	Coast of Galle, Sri Lanka	Fucoidan	AEC FTIR NMR	[54]
*Padina tetrastromatica*	Vizhinjam coast of Kerala, India	SPs	^1^H NMR DEAE-Cellulose chromatography	[55]
*Padina tetrastromatica*	Vizhinjam coast of Kerala, India	SPs	Elemental analysis UV-Vis GPC	[56]
*Padina tetrastromatica*	Coastal rocks of Mulloor, Vizhinjam, Thiruvananthapuram, Kerala, India	Sulfated fucan	HPTLC LC-ESI-MS DEAE-Cellulose chromatography	[57]
*Saccharina japonica*	Guemil-eup, Wando-gun, and Jeollanam-do, Republic of Korea	Fucoidans	Component analysis Elemental analysis FTIR UV-Vis XRD TGA TLC HPSEC-ELSD HPLC-ELSD	[58]
*Saccharina japonica*	Xiapu, Fujian province, China	Fucoidan	Component analysis HPSEC-MALLS-RID FTIR NMR GC	[59]
*Saccharina japonica* *Sargassum fusiforme Sargassum hemiphyllum* *Undaria pinnatifida*	Various	SPs	AEC HPSEC-MALLS-Visc-RID FTIR HPLC	[60]
*Sargassum binderi*	Hikkaduwa southern coast of Sri Lanka	-	IEC FTIR NMR	[61]
*Sargassum duplicatum*	Nhatrang Bay (Socialist Republic of Vietnam)	Fucoidan	Component analysis Chemical modifications ESI-MS/MS MALDI-TOF NMR HPSEC DEAE-Cellulose chromatography AGE	[62]
*Sargassum duplicatum* *Sargassum feldmannii*	Nhatrang bay (Socialist Republic of Vietnam)	Fucoidan	ESI-MS/MS NMR HPSEC Chemical modifications	[63]
*Sargassum horneri*	-	Fucoidan and sulfated fucooligosaccharides	NMR IEC PAGE	[64]
*Sargassum muticum*	Buarcos Bay (Figueira da Foz, Portugal)	Fucoidans	Component analysis ICP-OES HPLC-UV FTIR-ATR ^1^H NMR	[65]
*Sargassum pallidum*	Weihai, Yellow Sea, China	Fucoidans	Component analysis HPGPC-FTIR GC-FID	[66]
*Sargassum swartzii*	Coast of Kanyakumari, India	SPs	FTIR NMR UV-Vis TLC HPSEC TGA	[67]
*Sargassum wightii*	Tamil Nadu, India	-	Elemental analysis Component analysis FTIR TGA	[68]
*Turbinaria conoides*	Coast of Mandapam, Rameswaram, Gulf of Mannar, Tamil Nadu, India	Fucoidan	GPC HPLC NMR GC-MS DEAE-Cellulose chromatography Component analysis	[69]
*Turbinaria ornata*	Nhatrang Bay (Socialist Republic of Vietnam)	Fucoidan	DEAE ESI-MS/MS GC-MS NMR	[70]
*Turbinaria turbinata*	Malaysian origin	SPs	GC-FID FTIR HPSEC-MALS-RI DEAE-Cellulose chromatography NMR TGA	[71]
*Undaria pinnatifida*	Auckland, New Zealand	Fucoidan	Component analysis FTIR 2D-NMR HPLC-RID	[72]
**Rhodophyta (Red Algae)**				
*Chondrus canaliculatus*	Tunisian coasts, Sfax (“Sidi Mansour, Tabaroura”)	Fractions of SPs	Component analysis HPGPC FTIR-ATR HPLC-RID Solid-state ^13^C NMR	[73]
*Gelidiella acerosa*	Atlantic coast, Brazil (Búzios Beach, Nísia Floresta—Rio Grande do Norte)	SPs	Elemental analysis Component analysis FTIR NMR HPSEC	[74]
*Gelidium crinale*	Naozhou Island Sea, Zhanjiang City, Guangdong Province	SPs	Chemical modifications Component analysis FTIR HPLC-UV GPC	[75]
*Gigartina pistillata*	Collected at Spanish Atlantic coasts and obtained from a local supplier (Porto-Muiños, A Coruña, Spain)	Carrageenans	Component analysis FTIR HPSEC GC-FID	[46]
*Gracilaria caudata*	Brazilian Atlantic coast (Fleixeiras Beach, Trairí—Ceará)	SP	Component analysis GPC ICP-OES FTIR NMR	[76]
*Gracilaria caudata*	Northeast Atlantic coast of Brazil (Fleixeira Beach, Trairi—CE, Brazil)	SPs	FTIR NMR	[77]
*Gracilaria gracilis*	Wild Coast Abalone, East London, South Africa	SPs	Component analysis SEM-EDX FTIR GC-MS	[78]
*Gracilaria gracilis*	Dakhala shoreline, Morocco	Agars	FTIR NMR	[79]
*Gracilaria lemaneiformis*	Nan’ao Island of China	SPs	DEAE-Sephadex chromatography HPLC-ELSD FTIR GC-FID GC-MS	[80]
*Laurencia obtusa*	Coastal region of Bizerte (Tunisia) in the Mediterranean Sea	Complex SPs	Component analysis DEAE-Sephadex chromatography SEC-MALLS FTIR NMR	[81]
*Laurencia papillosa*	East-Mediterranean coastal waters of Lattakia, Syria	Carrageenans	Component analysis FTIR-ATR NMR GPC	[82]
*Osmundea pinnatifida*	Buarcos bay (Figueira da Foz, Portugal)	Agarans	Component analysis ICP-OES HPLC FTIR-ATR NMR	[65]
*Porphyra aitanensis*	Purchased from Pingtan Island, Fujian Province, China	SPs	HPLC-SEC-MALLS-RI UV EDS XRD	[83]
*Solieria filiformis*	Northeast Atlantic coast of Brazil (Flexeiras Beach, Trairi—Ceará)	SPs	HPSEC FTIR NMR Component analysis	[84]
**Chlorophyta (Green Algae)**				
*Caulerpa cupressoides* var. *flabellata*	Nísia Floresta, southern coast of Rio Grande do Norte, Brazil.	Sulfated galactans	Component analysis GPC NMR IEC	[85]
*Caulerpa lentillifera*	Cultivated, Dalian, Liaoning, China	SPs	Chemical modifications NMR GC-MS	[86]
*Caulerpa lentillifera*	Takalar, South of Sulawesi, Indonesia	SPs	FTIR HPLC NMR	[87]
*Caulerpa sertularioides*	Coast of Rio Grande do Norte, Brazil	SPs	Component analysis HPLC-RID GPC	[88]
*Chaetomorpha gracilis*	IMTA system Cinvestav Marine Station, Telchac	SPs	Component analysis FTIR NMR XRD TGA	[89]
*Codium isthmocladum*	Pirambuzios beach, Nisia Floresta, Rio Grande do Norte, Brazil	Sulfated homogalactans	AEC AGE GPC GC-MS NMR	[90]
*Gayralia brasiliensis*	Baía de Paranaguá, Paraná State, Brasil	Sulfated heterorhamnan	Component analysis HPSEC-MALLS-RI NMR	[91]
*Monostroma nitidum*	Coast of Yantai, China	SPs	Component analysis NMR GC-MS AEC HILIC-FT-MS FTIR HPGPC	[92]
*Monostroma nitidum*	Yellow Sea of China	Sulfated glucuronorhamnan	Component analysis FTIR NMR HILIC-FT-MS HPGPC RP-HPLC	[93]
*Ulva lactuca*	Taboulba and Sayada (Monastir—Tunisia)	Ulvans	Component analysis GC-FID HPSEC FTIR NMR	[94]
*Ulva lactuca*	Mediterranean Sea in Egypt (Alexandria in Abou Kir region)	SPs	Component analysis AEC FTIR HPLC-RID	[95]
*Ulva lactuca*	Wild Coast Abalone, East London, South Africa	SPs	Component analysis SEM-EDX FTIR GC-MS	[78]
*Ulva lactuca*	Ho-Ping Island, Keelung, Taiwan	Ulvans	Component analysis FTIR HPSEC	[96]
*Ulva lactuca* L.	Seashore of Nísia Floresta, RN, Brazil	SPs	Component analysis AGE FACE FT-Raman spectroscopy	[97]
*Ulva linza*	Lebanese Mediterranean coast	Ulvans	Elemental analysis Component analysis SEC HPLC FTIR NMR	[98]
*Ulva pertusa*	China	SPs	DEAE-Cellulose chromatography HPGPC GC FTIR AAS	[99]
*Ulva* sp.	Landrézac Beach, Sarzeau, Brittany, France	Ulvans	Component analysis HPSEC HPAEC MALDI-TOF	[100]
*Ulva* spp.	Swedish West coast	Ulvans	Elemental analysis FTIR SEC TGA SEM NMR HPAEC-PAD	[101]

## Data Availability

Data presented in this study are available on request from the corresponding author.

## Abbreviations

AAS	Atomic absorption spectroscopy
AEC	Anion-exchange chromatography
AGE	Agarose gel electrophoresis
CD	Circular dichroism
^13^C NMR	Carbon-13 nuclear magnetic resonance
2D-NMR	Two-dimensional nuclear magnetic resonance spectroscopy
DEAE-Cellulose	Diethylaminoethyl-Cellulose column chromatography
DEAE-Sepharose	Diethylaminoethyl-Sepharose column chromatography
EDS	Energy-dispersive X-ray spectroscopy
FACE	Fluorophore-assisted carbohydrate electrophoresis
FTIR	Fourier transform infrared spectroscopy
FTIR-ATR	Fourier transform infrared spectroscopy-attenuated total reflectance
GC-FID	Gas chromatography with flame ionization detection
GC-MS	Gas chromatography with mass spectrometry detection
GPC	Gel permeation chromatography
^1^H NMR	Proton nuclear magnetic resonance
HILIC-FT-MS	Hydrophilic interaction liquid chromatography-Fourier transform-mass spectrometry
HPAEC	High-performance anion-exchange chromatography
HPAEC-PAD	High-performance anion-exchange chromatography with pulsed amperometric detection
HPGPC	High-performance gel-permeation chromatography
HPLC-ELSD	High-performance liquid chromatography with evaporative light scattering detector
HPLC-RID	High-performance liquid chromatography with refractive index detection
HPSEC	High-performance size-exclusion chromatography
HPSEC-ELSD	High-performance size-exclusion chromatography with evaporative light scattering detector
HPSEC-MALLS	High-performance size-exclusion chromatography coupled with multi-angle laser light scattering
HPSEC-MALS-RI	High-performance size-exclusion chromatography-multi-angle light scattering and refractive index detection
HPSEC-UV-MALLS-RI	High-performance size-exclusion liquid chromatography with ultraviolet-multi-angle laser light scattering-refractive index detection
HPTLC	High-performance thin-layer chromatography
ICP-MS	Inductively coupled plasma-mass spectrometry
ICP-OES	Inductively coupled plasma-optical emission spectrometry
IEC	Ion-exchange chromatography
LC-ESI–MS/MS	Liquid chromatography-electrospray ionization-tandem mass spectrometry
MALDI-TOF-MS	Matrix-assisted laser desorption/ionization-time-of-flight mass spectrometry
MALLS	Multi-angle laser light scattering detection
RP-HPLC	Reversed phase-high-performance liquid chromatography
SDS-PAGE	Sodium dodecyl sulfate-polyacrylamide gel electrophoresis
SEC-MALLS	Size-exclusion chromatography-multi-angle laser light scattering
SEM	Scanning electron microscopy
SEM-EDX	Scanning electron microscope-energy-dispersive X-ray analysis
SLS/DLS	Static and dynamic light scattering
TGA	Thermogravimetric analysis
TLC	Thin-layer chromatography
UV-Vis	Ultraviolet-visible spectroscopy
XRD	X-ray diffraction
